# Productivity and global warming potential of direct seeding and transplanting in double‐season rice of central China

**DOI:** 10.1002/fes3.419

**Published:** 2022-08-31

**Authors:** Le Xu, Shen Yuan, Xinyu Wang, Guodong Yang, Pan Xiangcheng, Xing Yu, Fei Wang, Jianliang Huang, Shaobing Peng

**Affiliations:** ^1^ National Key Laboratory of Crop Genetic Improvement, Hubei Hongshan Laboratory, MARA Key Laboratory of Crop Ecophysiology and Farming System in the Middle Reaches of the Yangtze River, College of Plant Science and Technology Huazhong Agricultural University Wuhan Hubei China

**Keywords:** direct seeding, double‐season rice, greenhouse gas emission, yield performance

## Abstract

Labor and water scarcity requires crop establishment of double‐season rice to be shifted from traditional transplanting to direct seeding. Owing to the limited thermal time, only ultrashort‐duration cultivars of about 95 d can be used for direct‐seeded, double‐season rice (DDR) in central China. However, whether the shift in crop establishment of double‐season rice can reduce greenhouse gas emissions without yield penalty remains unclear. Field experiments were conducted in Hubei province, central China with three treatments of crop establishment in the early and late seasons of 2017 and 2018. Treatments included DDR with ultrashort‐duration cultivars (DDR_U_), transplanted double‐season rice with ultrashort‐duration cultivars (TDR_U_), or with widely grown cultivars which have short duration of about 110 d (TDR_S_). It was found that crop growth duration of DDR_U_ was 6–20 days shorter than that of TDR_U_ and TDR_S_, respectively. Ultrashort‐duration cultivars under DDR_U_ achieved 15.1 t ha^−1^ of annual yield that was 9.4% higher than TDR_U_, and only 3.2% lower than TDR_S_. DDR_U_ reduced the annual cumulative CH_4_ emission by 32.0–46.1%, but had no difference in N_2_O emission in comparison with TDR_U_ and TDR_S_. The highest CO_2_ emission was TDR_S_ followed by DDR_U_, and then TDR_U_. As a result, shifting from TDR_U_ and TDR_S_ to DDR_U_ decreased global warming potential and yield‐scaled greenhouse gas intensity by 28.9–53.2% and 20.7–63.8%, respectively. These findings suggest that DDR can be a promising alternative to labor‐ and water‐intensive TDR in central China that offers important advantages in mitigating agricultural greenhouse gas emissions without sacrificing grain yield.

## Graphical abstract

Direct‐seeded, double‐season rice (DDR) using ultrashort‐duration cultivars achieved 15.1 t ha‐1 of annual yield within 188 days.Yield performance of DDR was comparable to that of transplanted double‐season rice (TDR).Shifting from DDR to TDR significantly reduced GWP and GHG intensity by 28.9‐53.2% and 20.7‐63.8%, respectively.

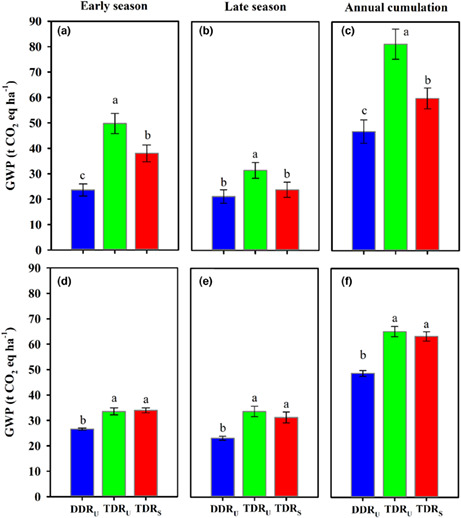

## 1 INTRODUCTION

Rice is the staple food for more than 65% population in China (Zhang et al., 2005). Since the scarcity of arable land per capita (i.e., 43% of the world average), double‐season rice that permits two harvests per year is an important cropping system to ensure sufficient food supply for nearly 1.4 billion people in China (Deng et al., 2019; Xu et al., 2020). However, the planting area of double‐season rice has reduced by about 6.2 million hectares from 1998 to 2019 (accounted for 20.5% of China's total rice planting area in 2019), causing a significant impact on food security (National Bureau of Statistics of China (NBSC), 2019). This change occurred because early‐ and late‐season rice crops in this system are manually transplanted into the puddled field and grown in flooded condition, in which large amounts of irrigated water and labor are consumed for crop establishment (Chauhan et al., 2017; Nie & Peng, 2017). As Chakraborty et al. (2017) and Fan et al. (2002) reported, exceeding 30% irrigation water for rice production is used in seedling nursery and field puddling and 25–50 person‐day ha^−1^ is required for rice transplanting. With rapid economic growth and urbanization in China, the productivity and sustainability of transplanted double‐season rice (TDR) is threatened by the emerging challenges of water and labor shortage for agriculture (Liu et al., 2014; Peng, 2014). The intensive resources consumption with low economic return has also reduced farmer's willingness to grow double‐season rice (Xu et al., 2018). Therefore, it is critical that double‐season rice cropping should be practiced in a sustainable intensification manner to save labor and water resources while maintaining high grain yield.

Direct seeding, referring to the process of sowing rice seeds directly into the field, has been proposed as an alternative to transplanting for reducing water and labor input, and gaining more economic profit (Farooq et al., 2011; Wang et al., 2022; Xu et al., 2019). At the beginning of the 21st century, there has been an increasing trend of shifting from rice transplanting to direct seeding in many countries including China (Pandey & Velasco, 2005; Sun et al., 2015). Hence, many researchers suggested that replacing seedling transplanting with direct seeding would offer the opportunity to the expansion of double‐season rice (Peng, 2014; Xu et al., 1995). However, shifting from TDR to direct‐seeded double‐season rice (DDR) is restricted by insufficient thermal time in central China, where only 190 to 209 days in a year are suitable for rice growing (Ai et al., 2014). This suggested that ultrashort‐duration cultivars matured within 95 days are required to be used for DDR. By comparison, rice cultivars with about 110 days growth duration are commonly used for TDR (RiceData, 2020). Shortening growth duration from 110 to 95 days for planting DDR might introduce the risk of yield loss due to substantial decline in the total amount of incident solar radiation during the growing period (Katsura et al., 2008; Yoshida, 1981). Our previous research has selected suitable ultrashort‐duration cultivars for DDR and identified plant traits associated with its high yield performance (Xu et al., 2018; Yu, 2016). But uncertainty still remains as to whether DDR using ultrashort‐duration cultivars can be as productive as TDR using short‐duration cultivars.

Rice‐based cropping regime, especially for double‐season rice, is a major emitter of long‐lived greenhouse gases (GHG) which accounted for about 30% and 11% of global agricultural methane (CH_4_) and nitrous oxide (N_2_O) emissions, respectively (Feng et al., 2013; Forster et al., 2007; IPCC, 2014). Meanwhile, shifts in rice cropping regime may provide the opportunities for mitigating GHG emission, considering the emission of these gases from rice fields is highly sensitive to crop management practices (Hao et al., 2022). It has been reported that the water‐saving direct‐seeded rice relative to flooded transplanted rice cropping regime has a high potential to suppress CH_4_ flux. For example, Wassmann et al. (2004) found that CH_4_ emission can be reduced by over 50% in direct‐seeded rice field conducting midseason drainages when compared to continuous flooding. However, the tradeoff relationship between CH_4_ and N_2_O fluxes often occurs due to water or other management practices (Gao et al., 2022; Xu et al., 2021), implying that crop management interventions targeting to decrease CH_4_ may be offset by the enhanced N_2_O emission (Zheng et al., 2000; Zou et al., 2005). Global warming potential (GWP) and greenhouse gas intensity (GHGI) are introduced to evaluate the tradeoff between the exchange of these gases and the comprehensive impact on climate change in the area and crop yield scale, respectively (Lashof & Ahuja, 1990; Qin et al., 2010; van Groenigen et al., 2013). Shifting from TDR to DDR will lead to the differences in agronomic practices such as rice variety, planting density, water regime, and crop phenology (Liu et al., 2014; Xu et al., 2018; Xu et al., 2022) which might alter GHG fluxes. However, to our knowledge, literatures comparing agricultural GHG emission between DDR and TDR cropping regimes are extremely limited, despite double‐season rice in central China is increasingly demanding direct seeding for saving the costs of labor and water.

Therefore, a two‐year field experiment was conducted in central China. The objectives of this study are to (a) determine whether DDR with the ultrashort‐duration cultivar can maintain the high yield performance in comparison with TDR; (b) quantify the climatic impact of the shift in double‐season rice cropping regime (from TDR to DDR) accounting of GWP and GHGI derived from CH_4_, N_2_O, and CO_2_ emissions, and thereby gain an insight into the potential of optimizing cropping regime arrangements in improving yield production and environmental sustainability.

## 2 MATERIALS AND METHODS

### 2.1 Experimental sites

**FIGURE 1:**
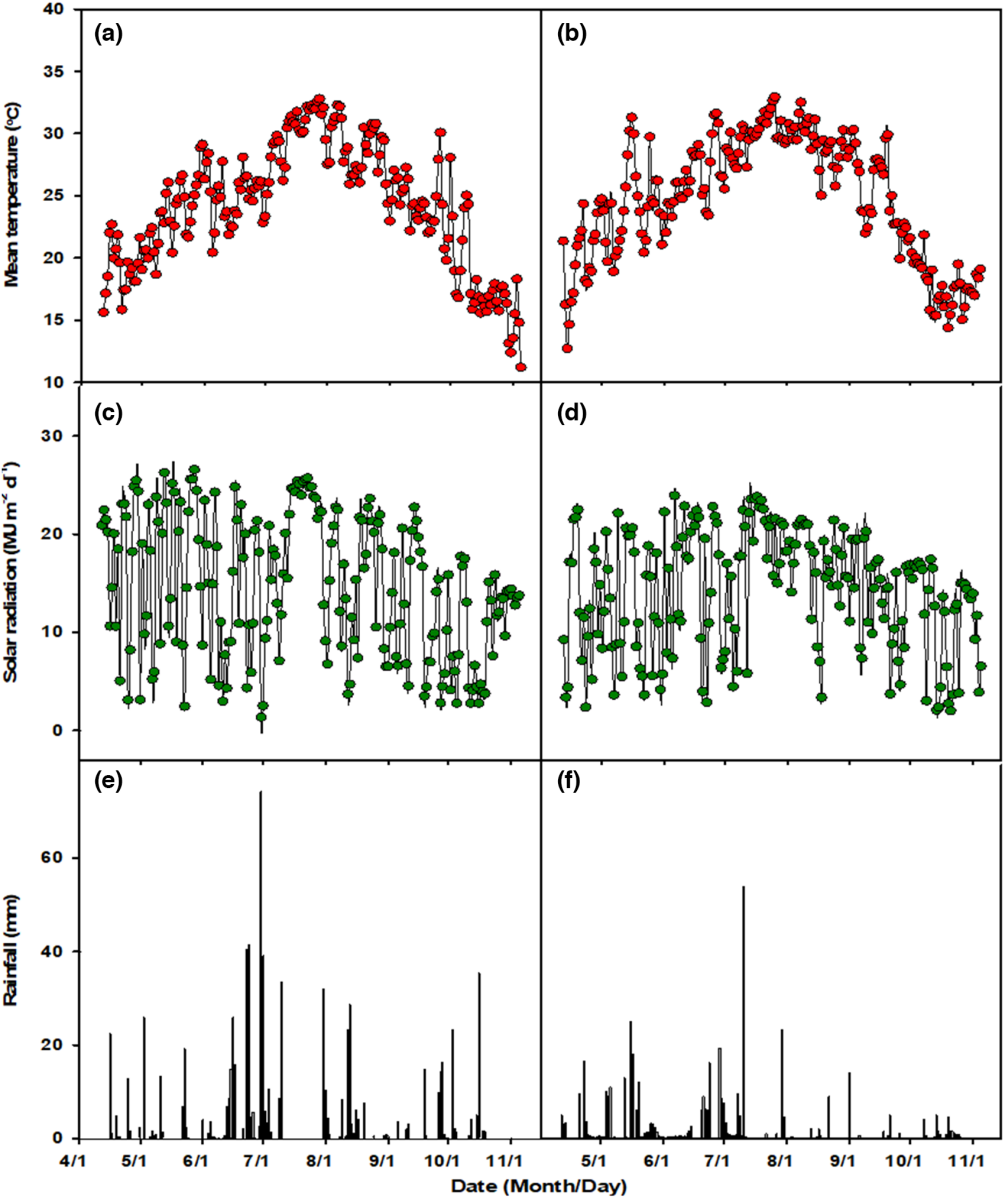
Daily mean temperature (a, b), solar radiation (c, d), and rainfall (e, f) during rice growing seasons in 2017 (a, c, e) and 2018 (b, d, f)

Field experiments were conducted in the subtropical environment of Wuxue county, Hubei province, central China (29° 51′N, 115° 33′E) in 2017 and 2018, which is a typical double‐season rice growing region. The mean daily solar radiation and temperature during the rice growing period across the two years were 14.5 MJ m^−2^ d^−1^ and 24.5°C, respectively. As presented in Figure 1, the temperature displayed an increasing trend in the early season but a decreasing trend in the late season from sowing to maturity, whereas the daily solar radiation did not show a consistent seasonal pattern. The total rainfall in 2017 and 2018 was 750 mm and 309 mm, respectively.

### 2.2 Experimental design and crop management

Experimental design was a randomized block design with four replications. The plot size was 25 m^2^ (5 × 5 m). Three treatments consisted of: (a) DDR using ultrashort duration cultivars (DDR_U_); (b) TDR using ultrashort‐duration cultivars (TDR_U_), and (c) TDR using widely grown cultivars with short duration of about 110 d (TDR_S_). The ultrashort‐duration cultivars, Xianzhaoxian 6 and Zaoxian 615, were used in both and TDR_U_ for the early and late seasons, respectively. These two varieties are indica inbreds that were bred for transplanted early‐season rice and could also be used in transplanted late‐season rice (Gong, 2012; Xia et al., 2010). In our previous experiments, these two varieties were identified as suitable for DDR owing to their ultrashort growth duration and good yield performance (Xu et al., 2018). Two widely grown elite cultivars, Ezao 18 and Liangyou 287, were used in TDR_S_ for the early and late seasons, respectively. EZ18 is an *indica* inbred and LY287 is a two‐line *indica* hybrid, both of which are bred for TDR. But their growth duration was not suitable for DDR due to insufficient thermal time in central China. The field and the treatment arrangement within the field were the same for the two seasons in both years.

For DDR_U_, the germinated seeds were manually broadcast into the puddled soil at a rate of 9 g seeds m^−2^ in the early season and 7 g seeds m^−2^ in the late season. Seeding was done on April 12 and July 21 in 2017, and on April 8 and July 19 in 2018 for the early and late seasons, respectively. For TDR_U_ and TDR_S_, pre‐germinated seeds were sown in a seedbed with the sowing date of March 25 and July 3 in 2017, and March 25 and July 1 in 2018 for the early and late seasons, respectively. In both years, 25‐d‐ and 20‐d‐old seedlings were transplanted for the early and late seasons, respectively at a hill spacing of 13.3 × 20 cm with three seedlings per hill. The fertilizer management was the same in both seasons and years. Nitrogen was applied weekly at 15 kg N ha^−1^ started at basal until heading because the N requirement of ultrashort‐duration cultivars has not been determined with precision previously. Weekly N application has been proved as a good fertilizer management for achieving high rice productivity with high fertilizer recovery efficiency (Peng & Cassman, 1998; Sheehy et al., 2000). The total N input was 120–135 kg ha^−1^ for early seasons and 90–105 kg ha^−1^ for late seasons in 2017 and 2018. Detailed information about N application can be found in Xu et al. (2022). Phosphorus as single superphosphate (31 kg P ha^−1^) was applied at the basal application. Potassium as potassium chloride was applied at the basal application (37 kg K ha^−1^) and panicle initiation (56 kg K ha^−1^). Before crop establishment, the field added with standing water was plowed and puddled to complete land preparation. For DDR_U_ plots, the soils were kept wet by controlling the irrigation after seed sowing to promote good establishment and then were flooded after the three‐leaf stage. A floodwater depth of 3–5 was maintained until one week before maturity except a drainage at maximum tillering stage to reduce unproductive tillers. For TDR_U_ and TDR_S_, the plots were kept flooded with a 3–5 cm water layer from transplanting to one week before maturity following the local farmer's water management. All bunds of plots were covered with a plastic film that was installed into a depth of 20 cm below the soil surface to minimize seepage between plots. Weeds, pests, and diseases were intensively controlled to avoid yield loss. Rice straw was completely removed from the paddy field during harvest.

### 2.3 Plant sampling and analysis

The dates of sowing, transplanting, panicle initiation, heading, and maturity were recorded for determining growth duration. Total growth duration refers to the period from seeding to maturity. Twelve hills for TDR_U_ and TDR_S_ and 0.5 m^2^ plants for DDR_U_ in each plot were sampled for growth analysis at panicle initiation, heading, and maturity. After recording stems number and panicles (when present), the plant samples were separated into leaves, stems, and panicles. The green leaf area was measured by a leaf area meter (LI‐3000; LI‐COR Inc., Lincoln, NE, USA) to determine the leaf area index (LAI). The dry weights of each organ were determined after drying at 70°C to constant weight, and then the aboveground biomass was calculated. At maturity, the panicles were hand‐threshed, and then the filled spikelets were separated from the unfilled spikelets by submerging them into tap water. The empty spikelets were separated from the half‐filled spikelets by sieving. Then, yield components including panicle number, spikelets per panicle, grain filling percentage, grain weight were measured, and harvest index was calculated. Grain yield was determined from a 5 m^2^ area in the center of each plot and was adjusted to 14% moisture content.

### 2.4 Gases sampling and analysis

CH_4_, N_2_O, and CO_2_ emissions from each plot were simultaneously measured at weekly intervals during rice growing seasons using the static closed chambers method starting from the fifth day after transplanting (Cha‐un et al., 2017). Static closed chambers were made of PVC that was equipped with two electric fans inside to ensure sufficient gas mixing and wrapped with a layer of sponge and aluminum foil outside to minimize air temperature excessive changes during gas sampling. Gas samplings were collected from 8:00 to 10:00 am. The chamber was placed and sealed on the fixed base that was buried in the field. Gas samples from the chamber headspace were collected at 0, 10, 20, and 30 min after chamber closure using 100 ml syringes fitted with three‐way stopcocks connected into the chamber. About 20 ml gas samples were transferred into pre‐evacuated vials with rubber stoppers. The air temperature inside the chamber was monitored during gas collection. The concentration of CH_4_, N_2_O, and CO_2_ in gas samples was analyzed in lab condition with gas chromatography (GC‐10 Plus; Shimadzu Scientific Instruments Inc., Kyoto, Japan). Gas fluxes for each plot were determined by the slope of change in the timeline‐based four samples. The cumulative GHGs emission was calculated according to the following formula:

$$\text{Cumulative emission}=\sum_{i=1}^{n}\left(\frac{R_{i}+R_{i+1}}{2}\times 24\times D_{i}\times 10^{-3}\right)$$

where *R*_*i*_ and *R*_*i* + 1_ are two consecutive days of GHG fluxes (mg m^−2^ h^−1^), and *D*_*i*_ is two adjacent sampling intervals (days).

The combined GWP derived from CH_4_, N_2_O, and CO_2_ emissions was calculated by adopting the IPCC factors. Thereafter, GHGI is calculated by dividing GWP by rice grain yield.

$$\mathrm{GWP}=25\times {\mathrm{CH}}_{4}+298\times N_{2}O+{\mathrm{CO}}_{2}$$

GHGI=GWP/grain yield

### 2.5 Soil sampling and analysis

Soil samples were taken from the upper 20 cm layer before experiment establishment and at the end of the last cropping season. Each sample of about 1.5 kg was a composite of five subsamples randomly taken within a plot. Plant detritus and any fragments were removed from soil samples after air‐drying at room temperature, then milled with a grinder to pass the 0.15 mm sieve. The milled samples were analyzed for pH, total soil N, Olsen phosphorus, exchangeable potassium, and soil organic carbon (SOC). The initial soil was a clay loam texture with a pH of 5.13, total N of 2.39 g kg^−1^, Olsen phosphorus of 54.3 mg kg^−1^, and exchangeable potassium of 140.7 mg kg^−1^. In addition, soil carbon sequestration (SCS) and the corresponding CO_2_ mitigation amount were preliminarily assessed for treatments of cropping regime by the SOC content change before and after two‐year field experiments according to the following formulas:

$$\mathrm{SCS}=\left({\mathrm{SOC}}_{\text{last}}-{\mathrm{SOC}}_{\text{initial}}\right)\times \mathrm{SW}$$

$${\mathrm{CO}}_{2}\;\text{mitigation amount}=\mathrm{SCS}\times \mathrm{CF}$$

where SOC_initial_ and SOC_last_ are the soil organic content (g kg^−1^) of the initial and last soil samples for each plot, respectively; SW is the topsoil weight that is assessed in the case of taking bulk density of 1.3, soil depth of 15 cm; CF is the conversion factor of CO_2_ emission from soil carbon (1 kg soil C = 3.664 kg CO_2_ eq).

Given that rice straw was completely removed from the field during harvest, the root‐derived carbon input was assessed to interpret the SOC difference between cropping regimes. Plant was destructively sampled to measure aboveground and root biomass at heading. Carbon concentration of root biomass was determined by Elementar vario MAX CNS/CN (Elementar Trading Co., Ltd, Germany). The root‐derived carbon input was calculated as the product of carbon concentration and root dry weight.

### 2.6 Statistical analysis

Analysis of variance was performed using Statistix 8.0 (Analytical Software, Tallahassee, FL, USA), and the means of treatments were compared based on the least significant difference (LSD) test at the 0.05 probability level (Katsura et al., 2008).

## 3 RESULTS AND DISCUSSIONS

### 3.1 Rice growth and yield performance

**FIGURE 2:**
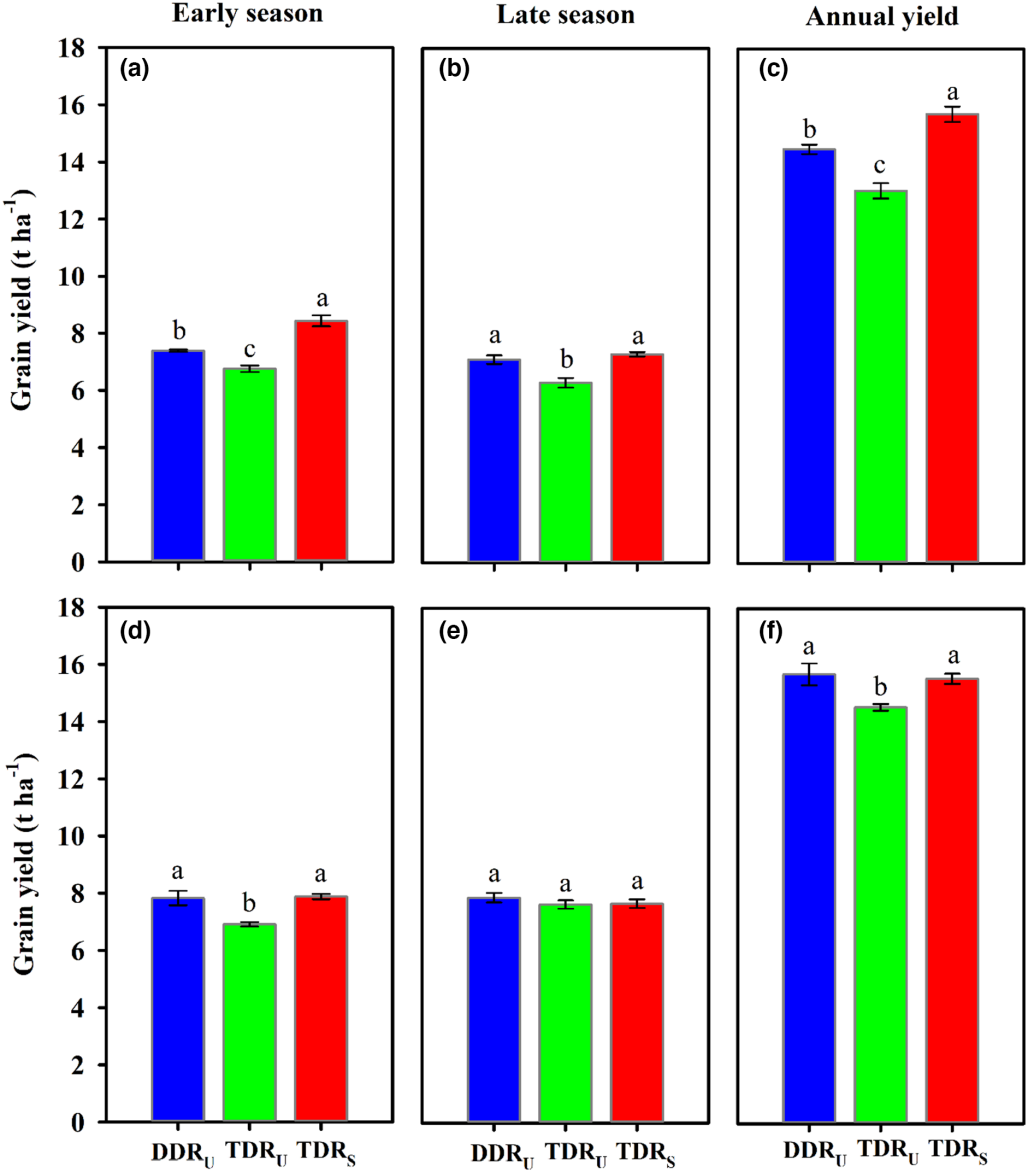
Grain yield performance of different cropping regimes in the early and late seasons of 2017 (a–c) and 2018 (d–f). Vertical bars represent ± SE of the mean. Different lower cases above the columns indicate significant differences among cropping regimes according to the LSD 0.05. DDR_U_, direct‐seeded, double‐season rice with ultrashort‐duration cultivars; TDR_U_, transplanted double‐season rice with ultrashort‐duration cultivars; TDR_S_, transplanted double‐season rice with short duration cultivar

DDR_U_ had growth durations within 95 days, whereas the growth duration of TDR_U_ and TDR_S_ ranged from 99 to 114 days across seasons and years (Table 2). On average, the growth duration of DDR_U_ was 10 days shorter than that of TDR_U_, and even 15 days shorter than that of TDR_S_. DDR_U_ using ultrashort‐duration cultivars achieved 15.1 t ha^−1^ of annual yield that was 9.4% higher than TDR_U_ when the same ultrashort‐duration cultivars were used, and only 3.2% lower than TDRS when the local elite cultivars were used (Figure 2). This annual yield was much higher than that of single‐season rice in the same region, suggesting the essential role of double‐season rice in the national food security (Yuan et al., 2017). Analysis of variance for yield‐related traits showed that year, season, cropping regime, and their interactions had a significant effect on grain yield, biomass production, and panicle number (Table 1). DDR_U_ produced 7.60 t ha^−1^ grain yield on average which was significantly higher than TDR_U_ except for the late season of 2018, and comparable to TDR_S_ except for the early season of 2017 (Figure 2). This yield level was consistent with previous studies in which double‐season rice yield in the same region ranged from 7 to 9 t ha^−1^ under optimum crop management and using short‐duration cultivars (Qin et al., 2013; Zhou et al., 2018). These results indicated that the change of cropping regime from traditional transplanted to labor‐ and water‐saving direct‐seeded double‐season rice would not result in significant yield penalty.

Grain yield variation between cropping regimes could be explained by biomass production at maturity rather than harvest index. Consistent with the yield performance, the biomass production of DDR_U_ was significantly higher than TDR_U_ except for the late season of 2018, and comparable to TDR_S_ except for the early season of 2017 (Table 2). Harvest indexes of cropping regimes ranged from 52.0% to 59.2% (Table 2) which was approached to the biological limitations. Khush (2001) stated that it is difficult to further increase rice harvest index after the Green Revolution, and the improvement of rice yield in recent decades has relied mainly on increasing biomass. Ultrashort‐duration cultivars under DDR_U_ significantly increased leaf area index and stem number at panicle initiation and heading as compared to TDR_U_ and TDR_S_ (Table 3). These early vigor characters benefited DDR_U_ to improve canopy intercepted radiation for photosynthesis, and thus reduce the negative impact of its shorter growth duration on biomass production. Sinclair and Horie (1989) and Laza et al. (2001) also emphasized the importance of early vigor characters on rice biomass. For yield components, DDR_U_ significantly increased panicle number, but reduced spikelets per panicle compared to TDR_U_ and TDR_S_ (Tables 1 and 2). No consistent difference in grain filling percentage and grain weight was observed among cropping regimes. As previous studies demonstrated, the increased panicle number was mainly responsible for the similar or even higher yield performance of direct‐seeded rice as compared with transplanted rice (Liu et al., 2014; Xu et al., 2019).

**TABLE 1:** Analysis of variance (ANOVA) for yield‐related and GHG emission traits

Traits	Year	Season	Cropping regime	Y × S^a^	Y × C	S × C	Y × S × C
Grain yield	*	**	**	**	**	**	*
Biomass	**	**	**	**	**	**	*
Harvest index	ns	**	**	ns	**	**	**
Panicles m^−2^	**	**	**	**	**	**	**
Spikelets panicle^−1^	ns	**	**	ns	**	**	**
Grain filling percentage	ns	ns	ns	ns	ns	**	ns
Grain weight	ns	ns	ns	ns	ns	ns	ns
CH_4_ emission	*	**	**	ns	**	ns	ns
N_2_O emission	*	*	**	*	ns	*	*
CO_2_ emission	ns	**	**	ns	**	**	*
GWP	**	*	**	*	*	**	*
GHGI	**	*	**	ns	ns	**	*

*Note*: ns denotes non‐significance at the 0.05 probability level, * denotes significant at *p* ≤ 0.05, and ** denotes significant at *p* ≤ 0.01.

^a^ Y × S: year × season, Y × C: year × cropping regime, S × C: season × cropping regime, and Y × S × C: year × season × cropping regime.

**TABLE 2:** Growth duration and yield attributes of cropping regimes in the early and late seasons of 2017 and 2018

Cropping regime	Growth duration (days)	Biomass (t ha^−1^)	Harvest index (%)	Panicle (m^−2^)	Spikelets (panicle^−1^)	Grain filling (%)	Grain weight (mg)
2017 early season							
DDR_U_	95	12.2 ± 0.1 b	57.3 ± 0.8 a	494 ± 6 a	74.8 ± 1.3 c	90.0 ± 0.4 a	21.1 ± 0.1 b
TDR_U_	105	11.0 ± 0.3 c	56.7 ± 0.6 a	369 ± 14 b	91.9 ± 1.2 b	86.8 ± 1.1 ab	21.3 ± 0.2 b
TDR_S_	113	13.1 ± 0.4 a	55.9 ± 0.5 a	336 ± 17 b	107.9 ± 1.2 a	84.5 ± 1.4 b	24.1 ± 0.1 a
2017 late season							
DDR_U_	93	12.5 ± 0.3 a	52.8 ± 1.2 b	437 ± 4 a	117.2 ± 1.5 a	75.7 ± 2.5 b	24.0 ± 0.1 a
TDR_U_	110	10.1 ± 0.2 b	52.0 ± 0.7 b	298 ± 5 c	82.5 ± 4.4 c	69.9 ± 1.0 c	22.1 ± 0.3 b
TDR_S_	110	11.3 ± 0.2 a	59.2 ± 0.3 a	356 ± 2 b	97.2 ± 1.4 b	85.5 ± 0.4 a	22.6 ± 0.1 b
2018 early season							
DDR_U_	94	13.0 ± 0.3 a	57.3 ± 0.8 a	540 ± 8 a	64.7 ± 1.8 c	90.8 ± 0.7 a	22.4 ± 0.2 b
TDR_U_	101	12.3 ± 0.2 b	56.7 ± 0.6 a	405 ± 7 b	96.8 ± 0.3 b	91.5 ± 0.4 a	21.1 ± 0.2 c
TDR_S_	114	14.6 ± 0.5 a	55.9 ± 0.5 a	335 ± 3 c	111.6 ± 1.7 a	79.0 ± 0.5 b	25.7 ± 0.1 a
2018 late season							
DDR_U_	93	13.9 ± 0.2 a	53.6 ± 0.5 a	482 ± 9 a	74.5 ± 1.6 b	88.8 ± 0.6 a	23.3 ± 0.3 a
TDR_U_	99	15.0 ± 0.4 a	53.1 ± 0.7 a	410 ± 11 b	111.8 ± 0.8 a	82.3 ± 0.9 b	21.1 ± 0.2 b
TDR_S_	99	14.1 ± 0.2 a	56.1 ± 0.7 a	381 ± 10 b	109.5 ± 3.4 a	88.6 ± 0.3 a	21.6 ± 0.1 b

*Note*: Within a column for each season, means followed by different letters are significantly different according to LSD 0.05. Values are presented in mean ± SE.

Abbreviations: DDRU, direct‐seeded, double‐season rice with ultrashort‐duration cultivars; TDR_U_, transplanted double‐season rice with ultrashort‐duration cultivars; TDRS, transplanted double‐season rice with short duration cultivars.

**TABLE 3:** Stem number per m^2^, leaf area index (LAI) at panicle initiation (PI) and heading (HD) stages, and plant height at maturity of cropping regimes in the early and late seasons of 2017 and 2018

Cropping regime	Stem number at PI	Stem number at HD	LAI at PI	LAI at HD	Plant height (cm)
2017 early season					
DDR_U_	947 ± 24 a	654 ± 26 a	3.28 ± 0.12 a	5.13 ± 0.19 a	80.8 ± 1.5 b
TDR_U_	478 ± 7 b	484 ± 21 b	1.67 ± 0.04 b	3.50 ± 0.13 b	77.4 ± 0.6 c
TDR_S_	378 ± 20 c	405 ± 12 c	1.48 ± 0.07 b	4.01 ± 0.14 b	91.9 ± 0.3 a
2017 late season					
DDR_U_	783 ± 23 a	472 ± 13 a	2.70 ± 0.08 a	4.60 ± 0.07 b	109.2 ± 0.7 a
TDR_U_	329 ± 16 b	423 ± 8 b	1.68 ± 0.07 c	4.53 ± 0.11 b	106.8 ± 1.0 b
TDR_S_	289 ± 13 c	381 ± 11 c	2.07 ± 0.05 b	5.11 ± 0.09 a	95.0 ± 0.3 c
2018 early season					
DDR_U_	745 ± 27 a	725 ± 18 a	3.05 ± 0.12 a	5.50 ± 0.12 a	73.1 ± 0.6 b
TDR_U_	358 ± 10 b	339 ± 6 b	1.80 ± 0.07 b	2.66 ± 0.05 c	74.1 ± 0.3 b
TDR_S_	282 ± 10 c	330 ± 17 b	1.57 ± 0.08 b	3.25 ± 0.10 b	88.8 ± 0.9 a
2018 late season					
DDR_U_	875 ± 43 a	626 ± 12 a	4.19 ± 0.05 a	6.28 ± 0.17 a	97.9 ± 0.9 c
TDR_U_	412 ± 19 b	413 ± 10 b	2.61 ± 0.07 c	5.56 ± 0.06 b	112.7 ± 0.7 a
TDR_S_	409 ± 10 b	391 ± 12 b	3.07 ± 0.07 b	5.55 ± 0.16 b	101.5 ± 0.9 b

*Note*: Within a column for each season, means followed by different letters are significantly different according to LSD 0.05. Values are presented in mean ± SE.

Abbreviations: DDR_U_, direct‐seeded, double‐season rice with ultrashort‐duration cultivars; TDR_U_, transplanted double‐season rice with ultrashort‐duration cultivars; TDR_S_, transplanted double‐season rice with short duration cultivars.

### 3.2 Effects of cropping regimes on greenhouse gas emissions

**FIGURE 3:**
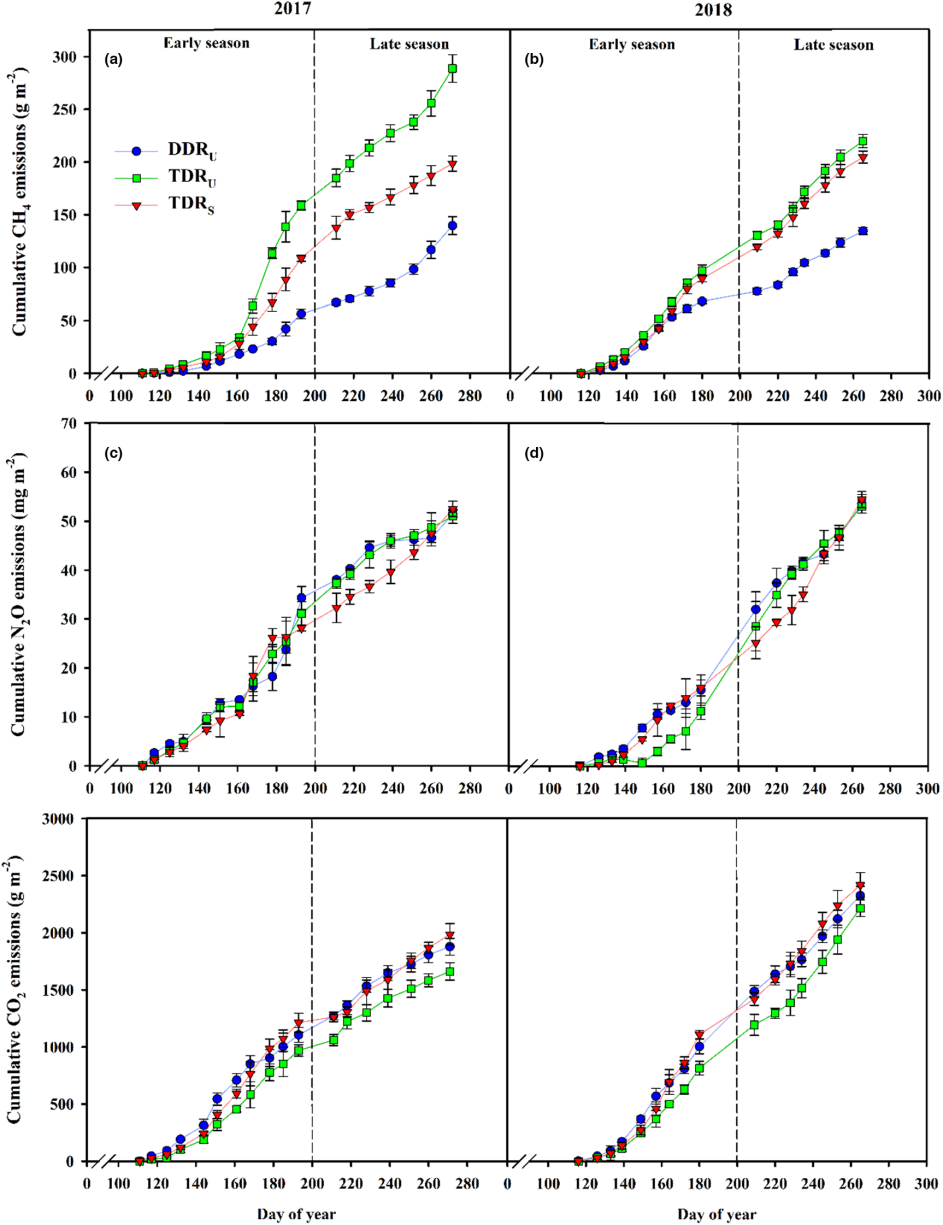
The cumulative CH_4_, N_2_O, and CO_2_ emissions from the different cropping regimes during rice cultivation in the early and late seasons of 2017 and 2018. Vertical bars represent ± SE of the mean. DDR_U_, direct‐seeded, double‐season rice with ultrashort‐duration cultivars; TDR_U_, transplanted double‐season rice with ultrashort‐duration cultivars; TDR_S_, transplanted double‐season rice with short duration cultivar

For evaluating the climatic impact of different cropping regimes, the cumulative CH_4_, N_2_O, and CO_2_ emissions during rice cultivation period were described in Figure 3. It was observed that the order of annual accumulated CH_4_ emission was TDR_U_ > TDR_S_ > DDR_U_. On average, DDR_U_ reduced accumulated CH_4_ emission by 32.0% compared to TDR_S_, and reduced by 46.1% compared to TDR_U_. The lower CH_4_ emission under DDR_U_ was mainly explained by reducing the period of submergence compared to long‐term flooding under TDR_U_ and TDR_S_ (Figures S1–S3). Many references have pointed out that changing a rice production system from anaerobic to aerobic can effectively suppress the activity of methanogenic bacteria during the degradation of organic carbon compounds (Adhya, Mishra, et al., 2000; Zhong et al., 2021). The cumulative CH_4_ emission was also significantly different between TDR_U_ and TDR_S_, and the difference in 2017 was larger than that in 2018 (Figure 3). This might be caused by the genotypic difference in plant growth characters. Overall, ultrashort‐duration cultivars under TDR_U_ increased tiller number by 18.0% in 2017 and 7.8% in 2018 than local elite cultivars under TDR_S_ at panicle initiation and heading stage (Table 3), which well supported the CH_4_ emission difference between TDR_U_ and TDR_S_, as well as the difference between 2 years. Aulakh et al. (2002) and Jalota et al. (2018) reported that the majority of CH_4_ was emitted through passing rice plant aerenchyma and out from the leaves, thereby tiller number was closely related to the methane transport capacity. Planting rice cultivars with fewer unproductive number has been recognized as an important agronomic practice to mitigate CH_4_ emission from rice field (Adhya, Bharti, et al., 2000).

DDR_U_ tended to increase N_2_O emission in comparison with TDR_U_ and TDR_S_ at the early growth stage (Figure 3 and Figure S2), which was in good agreement with the results of Liu et al. (2014) that the N_2_O emission of direct‐seeded rice was higher than that of transplanted rice due to the abundance oxygen in soil during midseason drainage. However, there eventually was no significant difference in annual accumulated N_2_O emission among cropping regimes. This was inconsistent with many studies in which midseason drainage led to a drop in CH_4_ but an increase in N_2_O emission in rice fields (Zhang et al., 2011; Zou et al., 2005). In this study, the high N_2_O emission of DDR_U_ at early growth stage should be offset by the reduced amount of soil mineral N. Considering fertilizer‐N application providing major mineral N source for N_2_O emission, early vigorous plant growth under DDR_U_ contributed to a 24% advantage in fertilizer‐N recovery efficiency over the TDR during rice growing period as demonstrated in our previous study (Xu et al., 2022), which thereby reduced N loss in the form of N_2_O emission. The split application of N at weekly interval also benefited rice crops to gain a high fertilizer‐N recovery efficiency that would largely reduce N_2_O emission, although this mode of N fertilization required the high labor input. Therefore, further research on DDR_U_ should be conducted to establish the optimum N management for achieving high grain yield and NUE with less labor input.

The seasonal pattern of CO_2_ fluxes and its cumulative emission clearly differed between cropping regimes which was determined by the trends of crop growth (Figure 3e,f; Figure S3). Previous studies have reported that CO_2_ emission from rice field was mainly originated from crop respiration and greatly affected by crop growth status (Cha‐un et al., 2017). The cumulative CO_2_ emissions of DDR_U_ were higher than those of TDR_U_ and TDR_S_ before heading, but the accumulated CO_2_ emissions at maturity followed the order of TDR_S_ > DDR_U_ > TDR_U_. This was well supported by the observations that crop growth of DDR_U_ was more vigorous expressed as rapid leaf area expansion and tiller production before heading compared to TDR_U_ and TDR_S_ (Table 3); but total biomass accumulation at maturity was TDR_S_ > DDR_U_ > TDR_U_ owing to the increased biomass production of TDR_S_ after heading (Table 2).

### 3.3 Effects of cropping systems on GWP mitigation and soil organic carbon stock

**FIGURE 4:**
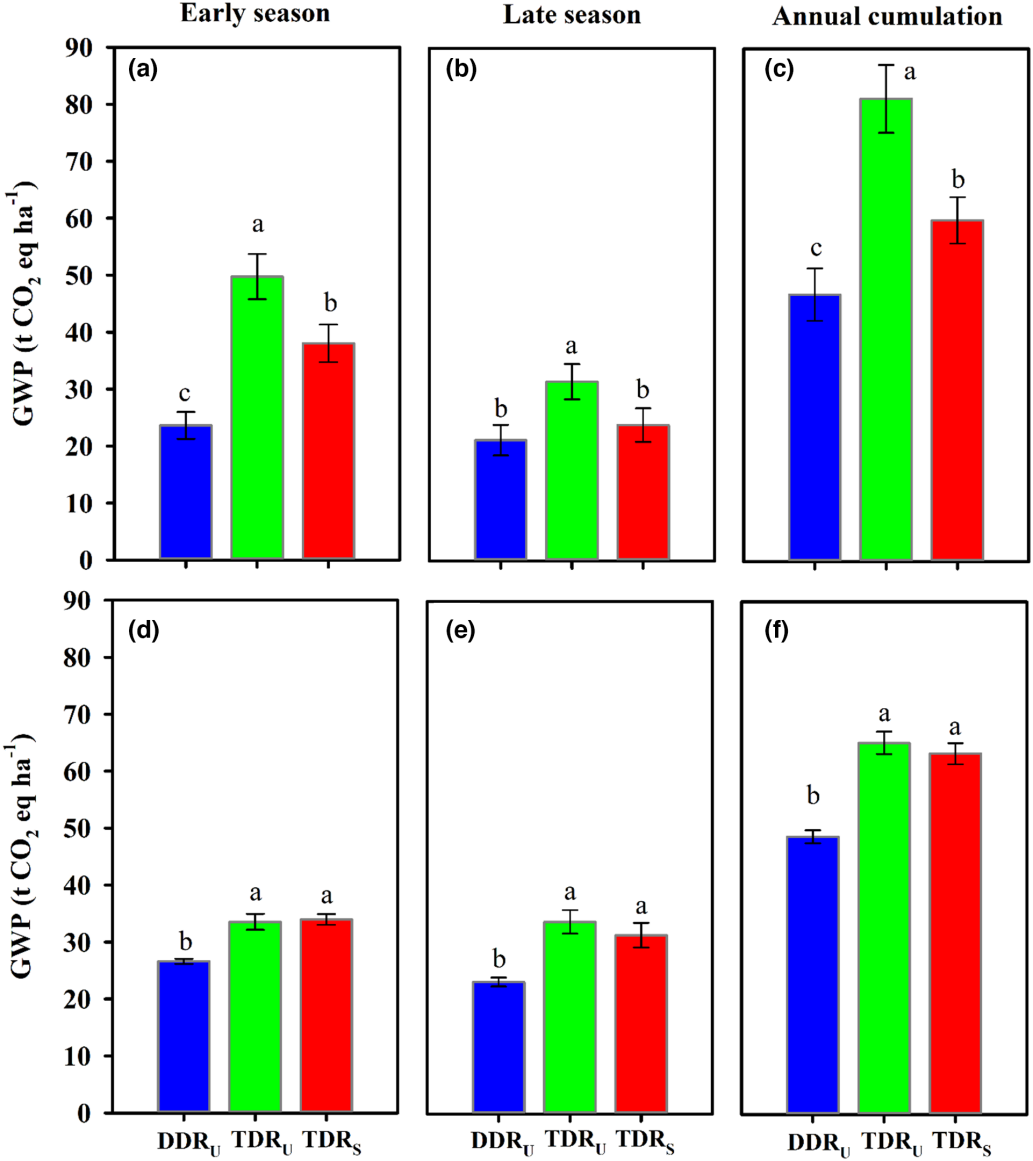
Seasonal and annual area‐scaled global warming potential (GWP) from the different cropping regimes during rice cultivation in the early and late seasons of 2017 (a–c) and 2018 (d–f). Vertical bars represent ± SE of the mean. Different lower cases above the columns indicate significant differences among cropping regimes according to the LSD 0.05. DDR_U_, direct‐seeded, double‐season rice with ultrashort‐duration cultivars; TDR_U_, transplanted double‐season rice with ultrashort‐duration cultivars; TDR_S_, transplanted double‐season rice with short duration cultivar

**FIGURE 5:**
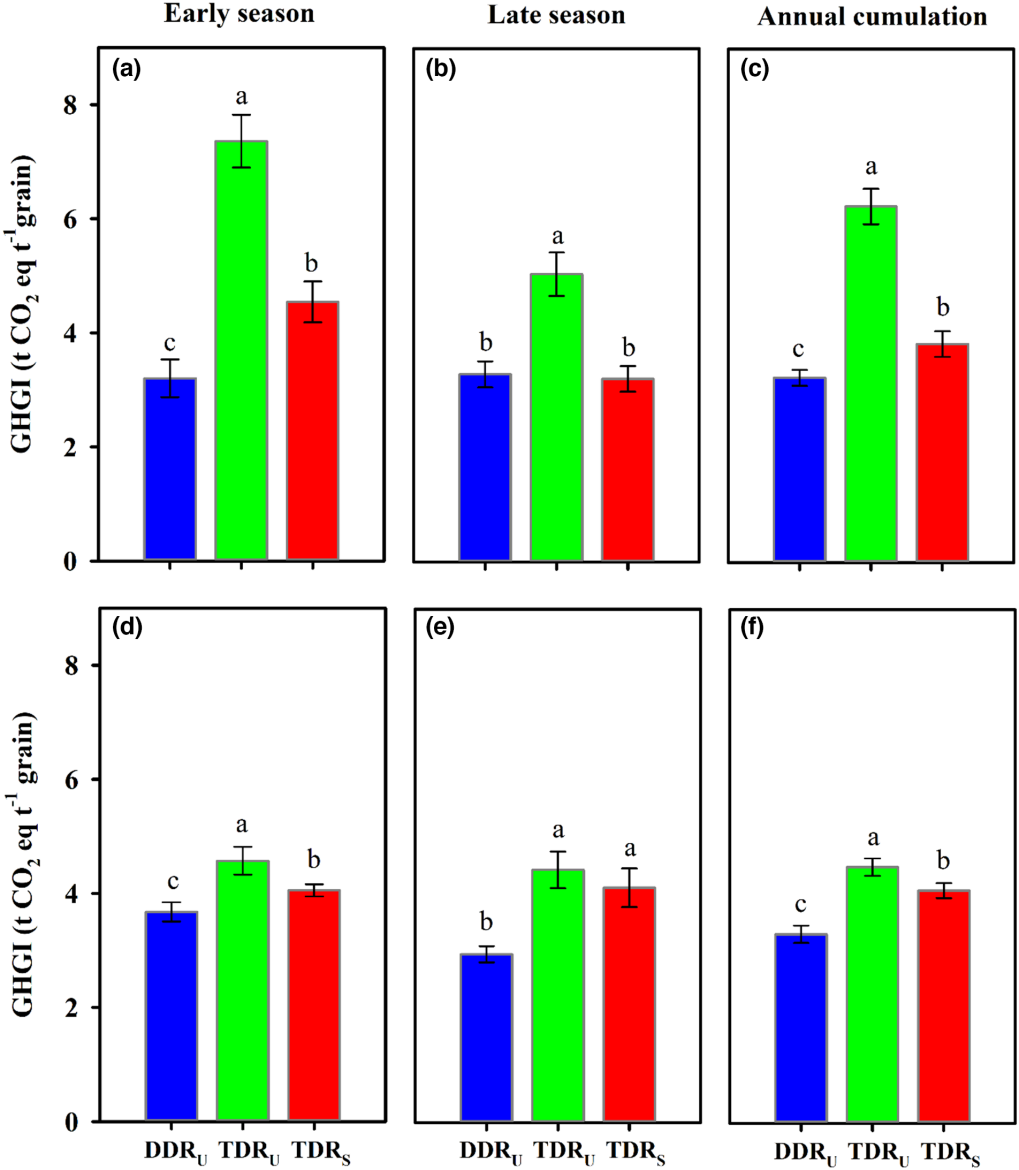
Seasonal and annual yield‐scaled greenhouse gas intensity (GHGI) of different cropping regimes during rice cultivation in the early and late seasons of 2017 (a–c) and 2018 (d–f). Vertical bars represent ± SE of the mean. Different lower cases above the columns indicate significant differences among cropping regimes according to the LSD 0.05. DDR_U_, direct‐seeded, double‐season rice with ultrashort‐duration cultivars; TDR_U_, transplanted double‐season rice with ultrashort‐duration cultivars; TDR_S_, transplanted double‐season rice with short duration cultivar

The area‐scaled GWP and yield‐scaled GHGI of annual CH_4_, N_2_O, and CO_2_ emissions were significantly affected by different cropping regimes (Table 1, Figures 4 and 5). Compared with TDR_U_ and TDR_S_, DDR_U_ decreased the GWP by 28.9–53.2% averaged across the 2 years, suggesting that direct‐seeded instead of transplanted double‐season rice cropping regime would largely mitigate the climatic impact derived from GHG emissions. Thereinto, the GWP was primarily contributed by CH_4_ emissions (60.8–80.8%), but less contributed by N_2_O and CO_2_ emissions (19.2–39.2%). This result was consistent with the finding of previous studies that direct‐seeded rice has lower GWP than traditional transplanted rice mainly due to the water‐saving irrigation reducing CH_4_ emission (LaHue et al., 2016; Liu et al., 2013). Since the comparable or higher annual yield was produced under DDR_U_, DDR_U_ reduced the yield‐scaled GHGI by 20.7–63.8% as compared with TDR_U_ and TDR_S_. In addition, rice paddy field can also sequestrate atmospheric CO_2_ into long‐lived soil pools and storing it securely, which is an essential part of strategy for mitigating the impacts on climate change. In the present study, positive SOC stock change was observed in double‐season rice cropping regimes within the consecutive two‐year cultivation, in which DDR_U_ sequestrated significantly higher carbon than TDR_U_ and TDR_S_ (Table 4). Witt et al. (2000) also reported that double cropping of rice resulted in a significant gain in soil carbon accumulation due to the substantial carbon input from crop residue and slow decomposition rate. Tang et al. (2018) measured the dynamic change of SOC at different soil depths during double‐season rice cropping in central China and reported the return of crop residue increased SOC by 10–26% at the soil depth of 0–20 cm, which was consistent to our results. The SOC stock difference between cropping regimes resulted from the higher residual root mass under DDR_U_ (Table 4). As such, the change from transplanted to direct‐seeded double‐season rice cropping regimes contributed to an increase of over 75% in CO_2_‐equiv. mitigation originated from the increased SOC stock.

**TABLE 4:** Soil organic carbon (SOC) and its change (△SOC) before and after 2‐year rice cultivation, soil carbon sequestration, and the corresponding CO_2_ mitigation amount for different cropping regimes

Cropping regime	Carbon input (t ha^−1^)^a^	Initial SOC (g kg^−1^)	Terminal SOC (g kg^−1^)	△SOC^b^ (g kg^−1^)	Carbon sequestration (t ha^−1^)	Mitigation of CO_2_ equiv. (t ha^−1^)
DDR_U_	6.17 ± 0.13 a	12.7 ± 0.2	16.4 ± 0.2	3.73 ± 0.33 a	7.27 ± 0.64 a	26.6 ± 2.3 a
TDR_U_	3.51 ± 0.15 c	12.7 ± 0.2	14.2 ± 0.2	1.44 ± 0.30 c	2.81 ± 0.58 c	10.3 ± 2.1 c
TDR_S_	4.46 ± 0.20 b	12.7 ± 0.2	14.8 ± 0.1	2.13 ± 0.22 b	4.16 ± 0.42 b	15.2 ± 1.5 b

*Note*: Means followed by different letters indicate significant differences according to LSD 0.05. Values are presented in mean ± SE.

Abbreviations: DDR_U_, direct‐seeded, double‐season rice with ultrashort‐duration cultivars; TDR_U_, transplanted double‐season rice with ultrashort‐duration cultivars; TDR_S_, transplanted double‐season rice with short duration cultivar.

^a^ The total amount of carbon input from root residues across the 2‐year experiment.

^b^ The change in SOC during rice cultivation is equal to terminal SOC minus initial SOC based on samples collected at the soil depth of 0–20 cm.

## 4 CONCLUSION

As labor and water scarcity is intensifying in China, a major shift in rice establishment is from traditional seedling transplanting to labor‐ and water‐saving direct seeding. This study explored the feasibility of practicing direct‐seeded, double‐season rice cropping in central China to ensure food security and mitigate agricultural GHG emission. Our results revealed that ultrashort‐duration cultivars under DDR_U_ achieved 15.1 t ha^−1^ of annual yield within 188 days. This annual yield was comparable to or even higher than TDR that used local elite cultivars or the same ultrashort duration. Compared to TDR, DDR_U_ reduced area‐scaled GWP by 41.0%, and reduced yield‐scaled GHGI by 42.2% as a result of the significant decline in CH_4_ emission. These results suggested that DDR with ultrashort‐duration cultivars has great potential to mitigate GHG emissions derived from rice cultivation without sacrificing grain yield, and thus it could be a promising alternative to traditional TDR cropping system in central China for saving agricultural labor and water input.

## FUNDING INFORMATION

No funding was received to support this research or manuscript.

## CONFLICT OF INTEREST

The authors have stated explicitly that there are no conflicts of interest in connection with this article.

## Supporting information


Figure S1–S3

## Data Availability

The data that support the findings of this study are available from the corresponding author upon reasonable request.
