# Supplementary material for: Productivity and global warming potential of direct seeding and transplanting in double‐season rice of central China
Source: Food Energy Secur. 2022 Aug 31;12(1):e419. doi: 10.1002/fes3.419 (PMC10078198; doi:10.1002/fes3.419)

Figure S1

Seasonal pattern of CH_4_ fluxes under different cropping regimes during rice cultivation in the early and late seasons of 2017 and 2018. Green arrows indicate the seed sowing and transplanting times, and yellow arrows indicate the harvest times. DDR_U_: direct-seeded, double-season rice with ultrashort-duration cultivars; TDR_U_: transplanted double-season rice with ultrashort-duration cultivars; TDR_S_: transplanted double-season rice with short duration cultivar.


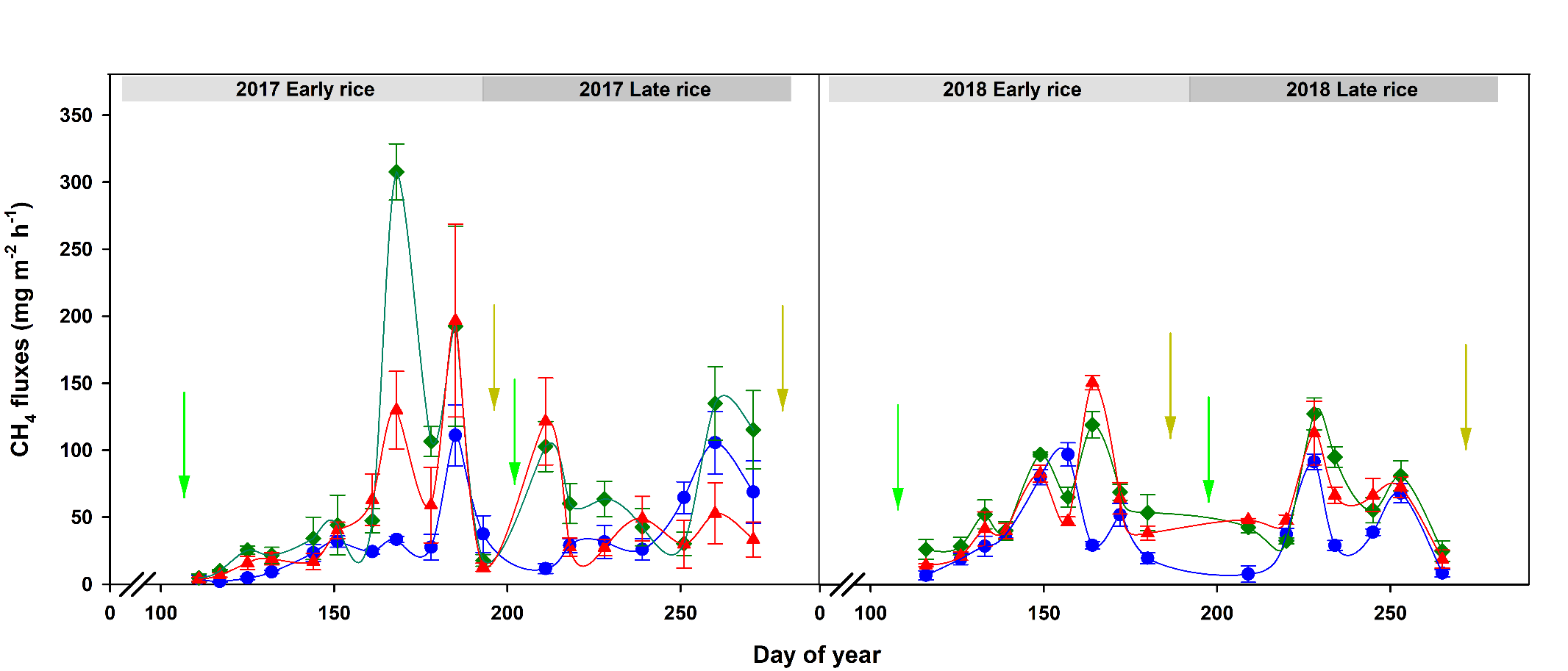


Figure S2

Seasonal pattern of N_2_O fluxes under different cropping regimes during rice cultivation in the early and late seasons of 2017 and 2018. Green arrows indicate the seed sowing and transplanting times, and yellow arrows indicate the harvest times. DDR_U_: direct-seeded, double-season rice with ultrashort-duration cultivars; TDR_U_: transplanted double-season rice with ultrashort-duration cultivars; TDR_S_: transplanted double-season rice with short duration cultivar.


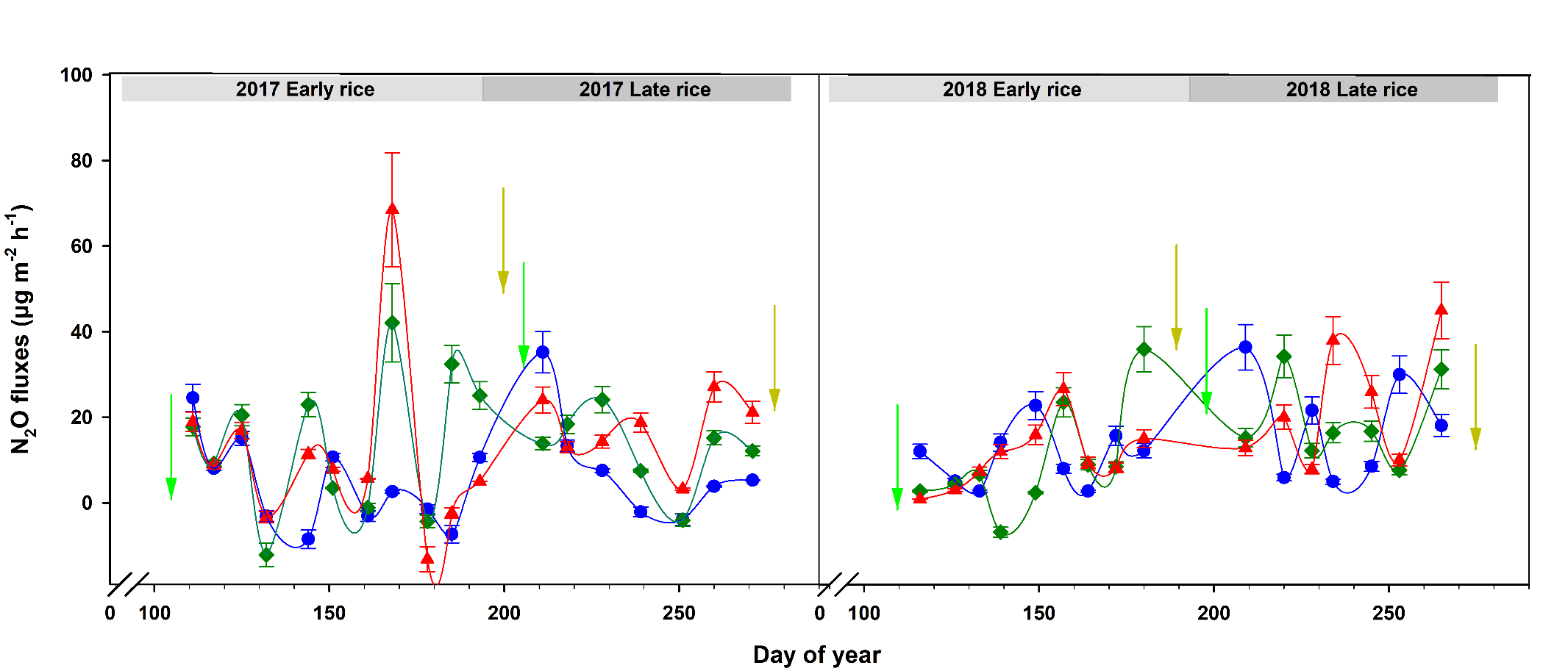


Figure S3

Seasonal pattern of CO_2_ fluxes under different cropping regimes during rice cultivation in the early and late seasons of 2017 and 2018. Green arrows indicate the seed sowing and transplanting times, and yellow arrows indicate the harvest times. DDR_U_: direct-seeded, double-season rice with ultrashort-duration cultivars; TDR_U_: transplanted double-season rice with ultrashort-duration cultivars; TDR_S_: transplanted double-season rice with short duration cultivar.


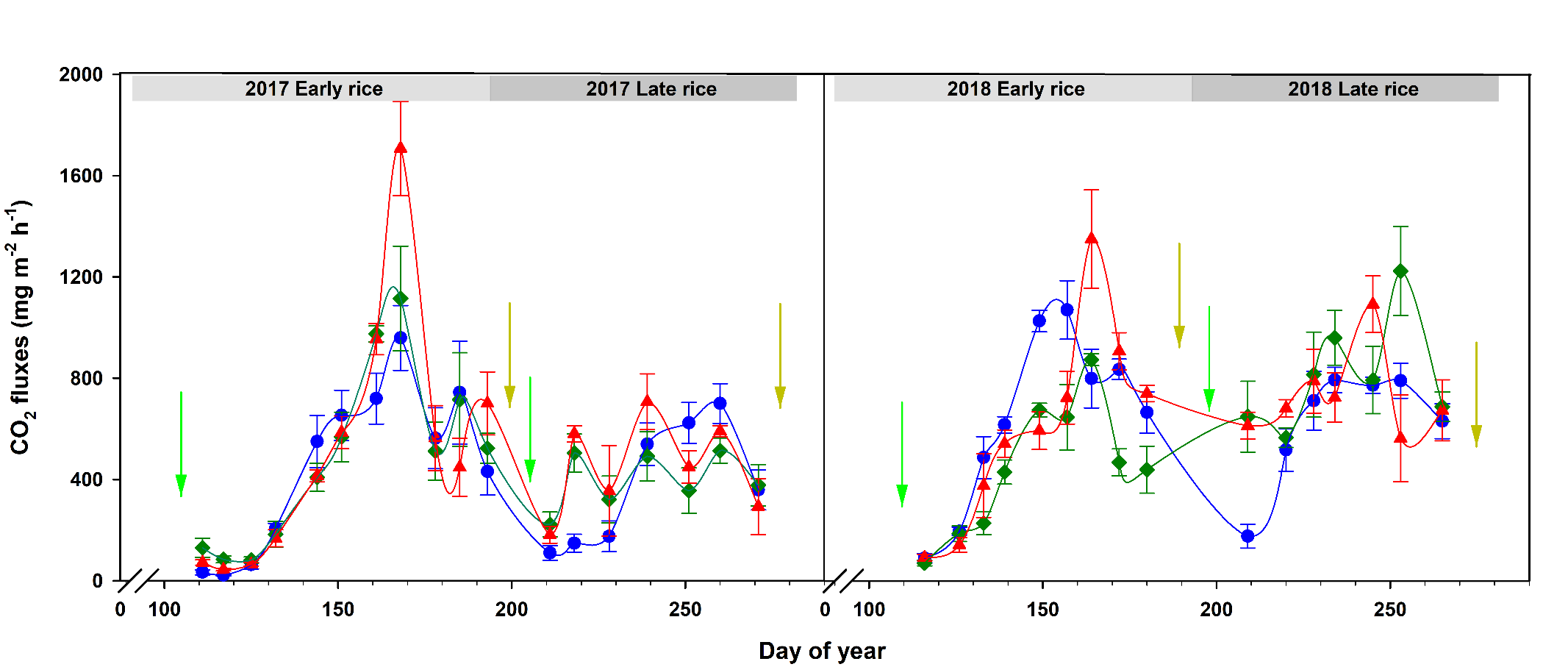

Supplement: Supplementary file 1 — Figure S1–S3 [file FES3-12-0-s001.docx]
